# Exploring the associations between behavioral health risk factors, abnormal eating attitudes and socio-demographic factors among Chinese youth: Survey of 7,984 vocational high school students in Hunan in 2020

**DOI:** 10.3389/fpsyt.2022.1000821

**Published:** 2022-11-07

**Authors:** Jieyu Liu, Ziwei Teng, Zirong Chen, Zirou Wei, Tianxiang Zou, Yue Qin, Hui Yuan, Minghui Liu, Jindong Chen, Hui Tang, Hui Xiang, Haishan Wu, Renrong Wu, Jing Huang

**Affiliations:** ^1^Department of Psychiatry, National Clinical Research Center for Mental Disorders, China National Technology Institute on Mental Disorders, The Second Xiangya Hospital of Central South University, Changsha, China; ^2^Department of Ultrasound Diagnosis, The Second Xiangya Hospital of Central South University, Changsha, China; ^3^Beijing Institute of Heart Lung and Blood Vessel Diseases, Beijing, China; ^4^Department of Stomatology, The Second Xiangya Hospital of Central South University, Changsha, China

**Keywords:** depression, anxiety, stress, behavioral health risk, abnormal eating attitudes, vocational high school

## Abstract

**Background:**

This study investigated the associations between behavioral health risk factors (anxiety, depression, stress, insomnia, drinking, smoking) and abnormal eating attitudes among Chinese vocational high school students (CVHSS). Potential moderating relationships were also explored with relevant socio-demographic factors of the student's age, sex, rural or urban community, household income, family type and educational level of the father and mother.

**Methods:**

A total of 7,984 students from three vocational high schools in Hunan, China completed a questionnaire about their socio-demographic characteristics, alcohol use, smoking, and symptoms of depression, anxiety, stress (21-item version of the Depression Anxiety Stress Scale), insomnia (8-item Athens Insomnia Scale), and abnormal eating attitudes (19-item Chinese version of Eating Attitudes Test).

**Results:**

The prevalence rates for behavioral health problems among these students ranged widely depending on the risk factor: 42.5% insomnia, 41.3% anxiety, 26.2% depression, 14.4% stress, 13.7% drinking, and 8.3% smoking. Additionally, 61.7% of students were at-risk for at least one of these six behavioral health disorders. Abnormal eating attitudes were associated with depression (*r* = 0.422), anxiety (*r* = 0.490), stress (*r* = 0.490), and insomnia (*r* = 0.375), with all of these relationships being significant (*p* < 0.01) and large size statistical effects. However, other analyzes found that none of the socio-demographic background factors had meaningful associations with the behavioral health risk factors (0 of 28 tests) and very few background factors were associated with the abnormal eating attitude measures (only 3 of 35 tests). Females had higher levels than males on dieting and bulimia but not on the other two eating attitude components.

**Conclusions:**

This study determined that behavioral health risk factors (sleep problems and anxiety in particular) were common among high school students in China and that mental health and sleep disorder risks also tended to co-occur in some students with abnormal eating attitudes. Therefore, prevention and early identification programs for behavioral risk factors are needed for this population. It is important to pay more attention to students with abnormal eating attitude-related symptoms, who may have also underlying mental health problems and need further evaluation.

## Introduction

According to data released by the World Health Organization, 13% of the global population aged 10−19 years have mental health problems, such as depression, anxiety, and stress, due to multiple physical, emotional, and social changes (1). These mental health problems may result in poor academic performance, lack of communication with friends and family, substance abuse, and even suicide ideation (2–4). However, most cases have not been detected and treated since adolescents tend not to seek help for mental health problems because of concerns about privacy and being ridiculed by others (5). Worse still, these circumstances may continue to damage their physical and mental health and limit their chances of leading a fulfilling life in adulthood. Therefore, it is imperative to detect and treat mental health problems in adolescents in a timely manner.

Several studies report that depression, anxiety, and stress were associated with abnormal eating attitudes (6–9). Le Grange et al. (10) found that depression was one of the developmental correlates of abnormal eating attitudes in an Australian population aged 15–16 years. Furthermore, in a group of female medical undergraduate students in Malaysia, depression was reported to be significantly positively correlated with abnormal dietary attitudes and negatively correlated with body image (11). Goel et al. (12) reported that depression and anxiety mediate the relationship between insomnia and abnormal eating attitudes in college women. The study conducted by Drieberg et al. (13) suggested that in clinical populations of adolescents, anxiety, and depression mediate the relationship between perfectionism and abnormal eating attitudes. In addition, Okumus et al. (14) found that job stress significantly positively influences both emotional and external eating among 372 hotel employees in Antalya.

Although the above studies have explored the association of depression, anxiety, and stress with abnormal eating attitudes in different populations (15), to the best of our knowledge, no study has focused on a youth group in China; specifically, Chinese vocational high school students (CVHSS), who constitute a huge demographic with more than 16 million students currently enrolled (16). After graduating from junior high school, Chinese students usually have two choices. The first is that students with better academic performance often choose to attend regular high schools to prepare for college. Another is that students with lower academic performance are more likely to choose vocational high schools and start working directly after graduation. Because of the significant differences in family relations, academic performance, social and cultural background, and other factors, CVHSS are more prone to mental health problems than ordinary high school students (17). Therefore, the present study aimed to explore the associations between behavioral health risk factors (anxiety, depression, stress, insomnia, drinking, smoking), abnormal eating attitudes and socio-demographic factors among CVHSS. Specific research questions included: (1) prevalence of common behavioral health risk factors, (2) associations of socio-demographic factors with behavioral health risk factors, (3) scores of abnormal eating attitudes, (4) associations of socio-demographic factors with abnormal eating attitudes, and (5) associations of behavioral health risk factors and abnormal eating attitudes.

## Method

### Participants

This study adopted a cross-sectional design. Through convenience sampling, we recruited 8,213 students (2,452, 3,994, and 1,767 students) from three local vocational high schools in Hunan, China, from October to December 2020.

### Informed consent and ethics

The study proposal was approved by the Ethics Committee of Xiangya Second Hospital of Central South University, Hunan, China. Participants over 18 signed a formal informed consent form before participation. In the case of minors, informed consent was obtained from a parent and/or legal guardian. Informed consent and parent/guardian informed consent are given in Informed Consent Materials in Appendix S1.

### Description of the sample

Participants completed the questionnaires with the assistance of teachers, and the questionnaires were presented in Chinese. The questionnaires followed the principle of anonymity and voluntary participation. All participants were informed of their right to decide whether to participate and that they could refuse or withdraw participation at any time. All methods were performed in accordance with the relevant guidelines and regulations. Of 8,213 Participants, 229 students declined, resulting in a response rate of 97.21% (*n* = 7,984). Of 7,984 total sample of completed questionnaires, 47.4% (3,788/7,984) were 16 years old and 49.7% (3,965/7,984) were female. Table 1 shows the socio-demographic characteristics of the participants.

**Table 1:** Socio-demographic characteristics of participants.

**Variable**	**Number**	**Percent (%)**
**Age:**		
≤15 years old	1,812	22.7
16 years old	3,784	47.4
17 years old	1,917	24
≥18 years old	471	5.9
**Sex:**		
Male	4,019	50.3
Female	3,965	49.7
**Community:**		
Rural	6,661	83.4
Urban	1,323	16.6
**Household income/year (yuan):**		
<1,00,000	6,479	81.1
≥1,00,000	1,505	18.9
**Education level—father:**		
Junior middle school and below	5,362	67.1
High school or technical school	2,403	30.1
College or university and above	219	2.8
**Education level—mother:**		
Junior middle school and below	5,616	70.3
High school or technical school	2,199	27.5
College or university and above	169	2.1
**Family type:**		
Nuclear family	4,261	53.4
Single parent family	1,111	13.9
Reorganized family	656	8.2
United family	1,126	14.1
Other types	830	10.4

N = 7,984.

### Clinical measures

#### Socio-demographic measures

The participants' socio-demographic characteristics included age (how old are you? Options: ≤ 15, 16, 17, or ≥18 years old), sex (what is your sex? Options: male or female), community (where are you from? Options: urban or rural), household income/year (what is your household income/year? Options: <1,00,000 or ≥1,00,000 yuan), parents' education level (What are your parents' education levels? Options: middle school and below, high school or technical school, or college or university and above), and family type (what is your family type? Options: nuclear family, single-parent family, reorganized family, united family, or other types).

#### Behavioral health risk measures

The 21-item version of the Depression Anxiety Stress Scales (DASS-21) was used to assess the severity of participants' symptoms of depression, anxiety, and stress, which includes seven items for each subscale of depression, anxiety, and stress, such as a depression sample item: “I felt down-hearted and blue,” an anxiety sample item: “I was worried about situations in which I might panic and make a fool of myself,” and a stress sample item: “I found it difficult to relax” (18). Participants were asked to rate the degree of each symptom experienced in the past week on a 4-point Likert-type scale. The rating scale is as follows: 0: did not apply to me at all, 1: applied to me to some degree, or some of the time, 2: applied to me to a considerable degree or a good part of time, and 3: applied to me very much or most of the time. The scoring cutoffs for the level of clinical risk were used from (19), which first required multiplying the summary scale scores by 2. The scores of moderate or higher on each scale were considered as clinical risk status (20). These three scales were all significantly correlated with each other (*r* ranged from 0.82 to 0.86).

The Athens Insomnia Scale (AIS) was adopted to assess the symptoms of insomnia in participants by evaluating eight factors; of these, the first five factors are related to nocturnal sleep, and the rest are related to daytime dysfunction (21). The score range of these factors is 0–3 and sleep was evaluated based on the cumulative score of all factors. A cumulative score >6 on the AIS indicated insomnia. The reliability of AIS has been confirmed in the Chinese population (22).

In addition, two other behavioral health risk factors were recorded, including smoking (do you have a smoking habit? yes or no) and drinking (do you have a drinking habit? yes or no).

#### Abnormal eating attitudes

The Chinese version of the Eating Attitudes Test (EAT-19), a revised version of EAT-26 (23), was used to assess the severity of participants' abnormal eating attitudes, such as dieting (eight items), bulimia and food preoccupation (four items), awareness of food contents (four items), and compensatory behavior (three items) (24). The scale has 19 self-report items and the score range of each item ranges from 1 to 6 (i.e., “Never” = 1, “Rarely” = 2, “Sometimes” = 3, “Often” = 4, “Very Often” = 5, and “Always” = 6). Note that this scale did not provide the scoring cutoffs. The higher the total score of all items, the more the participants' eating attitude deviates from normal. These four scales were all significantly correlated with each other (*r* ranged from 0.49 to 0.66).

As shown in Table 2, all of the multi-item subscales and full scales had acceptable levels of internal reliability (alpha range from 0.69 to 0.92) (25).

**Table 2:** Psychometric properties of measures of behavioral health risks and abnormal eating attitudes.

**Variable**	**Number of items**	**Rating Range**	**Reliability Alpha**	**Average rating *M (*SD)**	**Percent at risk (%)**
**Behavioral health problems**					
Mental health total	21	0–126	0.92	28.33 (24.97)	43.9
Anxiety	7	0–42	0.80	9.10 (8.47)	41.3
Depression	7	0–42	0.83	8.95 (8.79)	26.2
Stress	7	0–42	0.82	10.28 (8.95)	14.4
Insomnia	8	0–24	0.81	6.23 (4.00)	42.5
Drinking	1	–	–	–	13.7
Smoking	1	–	–	–	8.3
**Abnormal eating attitudes** ^a^					
Dieting	8	0–48	0.87	18.98 (8.93)	–
Bulimia and food preoccupation	4	0–24	0.85	8.09 (4.60)	–
Awareness of food contents	4	0–24	0.69	6.69 (4.05)	–
Compensatory behavior	3	0–18	0.75	4.28 (2.76)	–
Total scale	19	0–114	0.88	38.04 (17.87)	–

N = 7,984.

^a^Prevalence rates among CVHHS for abnormal eating attitudes cannot be presented since this scale did not provide the scoring cutoffs.

#### Total number of health risks

The total number of risks per student across the six behavioral health risks (range 0–6) and their total score for abnormal eating attitudes (range 0–114) were also recorded.

### Statistical analysis

SPSS (version 26.0, Chicago, Illinois) was used to analyze the data. Kolmogorov-Smirnov one-sample test was used to examine the normal distribution of the continuous data variables. Results found that each of the continuous data behavioral risk and eating attitude measures did not conform to the normal distribution. This skew in the data was expected, however, considering the sample included mostly healthy high school students, with only a minority having certain risk factors. Alternative testing procedures were also conducted using the Mann-Whitney *U*-test to explore the associations between behavioral health risks and eating attitude measures. These results indicated a similar pattern of results as presented in the paper using other correlational analytic procedures. The details of these alternative tests are not shown but are available upon request. Correlation analysis between two continuous variables or between continuous variables and ordered rank variables was performed using Spearman's correlation. The correlation *r* statistic created from the Chi-square test was used for correlation analysis between two classification variables. Point biserial correlation *r* value was used to show the correlation between binary and continuous variables.

Given the very large sample size involved, we had a level of 0.99 for the statistical power to detect a significant difference beyond chance. Therefore, even very small and practically meaningless differences could be declared “significant” in this context. Thus, all tests were presented as a standardized statistical effect size metric. all tests need to also present a standardized statistical effect size metric. Based on a recent meta-analysis review by (26) the terms small, medium, and large correspond to correlations of *r* = 0.10, 0.20, and 0.30. Findings in the study are interpreted using this effect size criterion of at least a small size result, regardless of if the result was significant at *p* < 0.05 level.

As the data was derived from three schools, the school site factor was examined as a potential moderating factor. The results indicated only trivial effect sizes or non-significant findings when comparing the three schools on the socio-demographic factors, behavioral health risks, and attitudes toward eating. The details of these tests are not shown but are available upon request.

## Results

### Prevalence rates among CVHHS for common behavioral health risk factors

**Figure 1:**
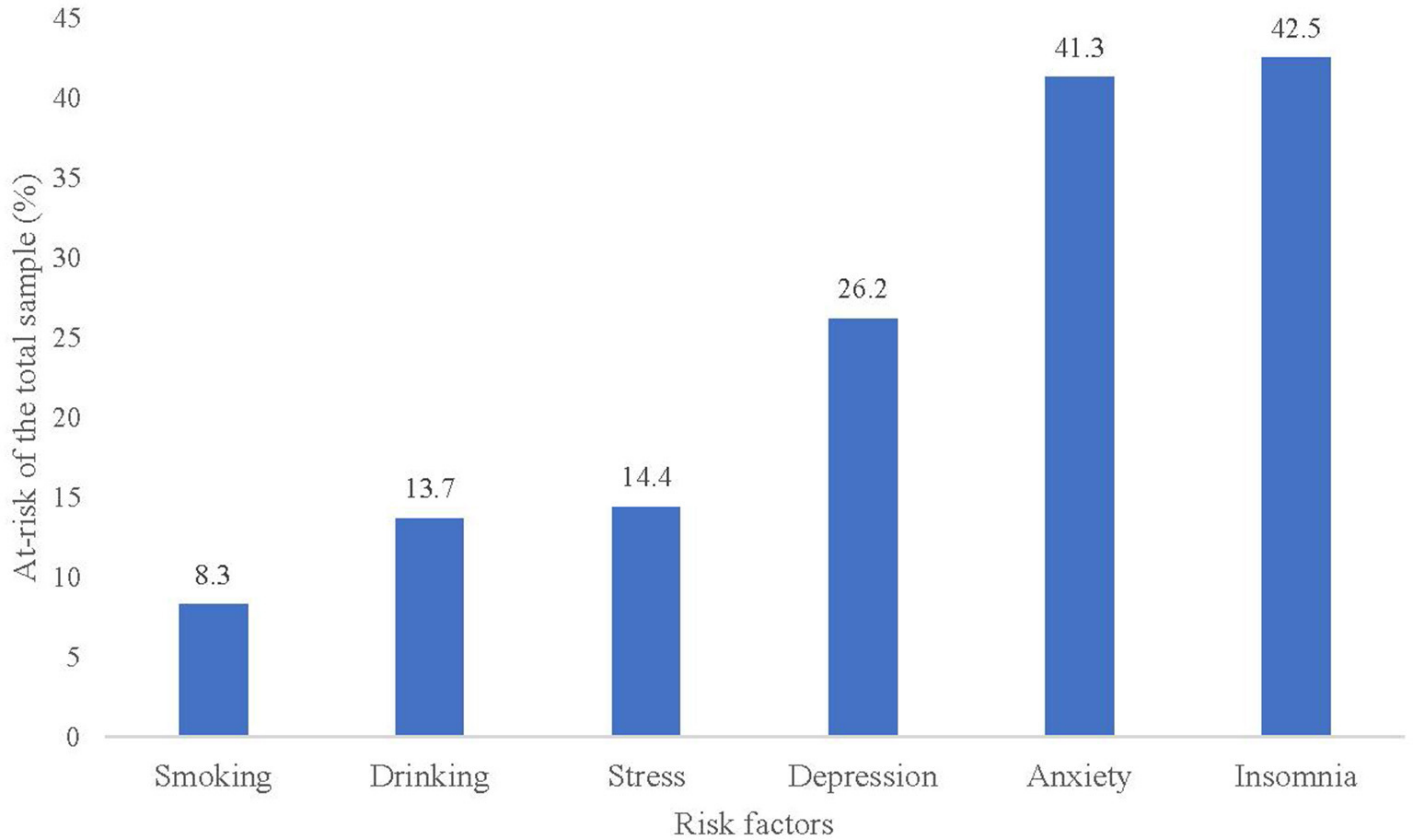
Prevalence of behavioral health risk factors among CVHSS.

**Figure 2:**
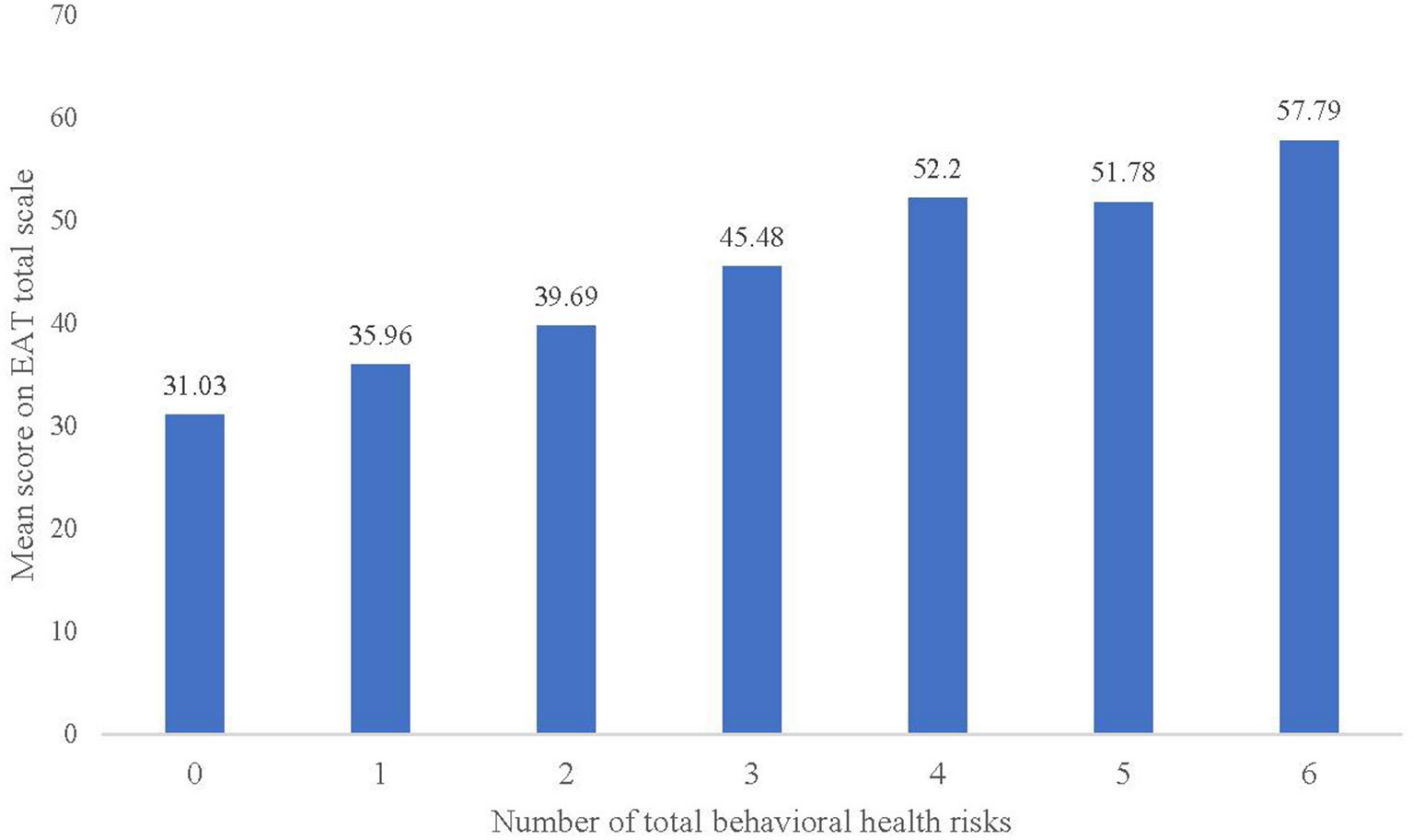
Mix of the number of total behavioral health risks per person among CVHSS.

Of the participants, 43.9% (3,512/7,984) had mental health problems (at least one symptom of depression, anxiety, and stress). 42.5% (3,393/7,984) had insomnia symptoms, 41.3% (3,297/7,984) had anxiety symptoms, 26.2% (2,092/7,984) had depression symptoms, 14.4% (1,149/7,984) had stress symptoms, 13.7% (1,090/7,984) drank before, and 8.3% (665/7,984) smoked before (see Table 2 and Figure 1). The percentage of participants at each level of clinical severity for anxiety, depression and stress (% at risk) is presented in Table 3. Moreover, from Figure 2, we found that 61.7% (4,926/7,984) of participants had at least one behavioral health risk, and even 0.9% (72/7,984) had all six behavioral health risks.

**Table 3:** Percentage of students at each level of clinical severity on the three measures of mental distress.

**Measure**	**Severity level on DASS-21**				
	**Normal**	**Mild**	**Moderate**	**Severe**	**Extremely severe**
Anxiety	50.1	8.6	22.4	7.4	11.5
Depression	60.0	13.8	16.9	4.3	5.0
Stress	76.9	8.7	7.5	4.2	2.7

N = 7,984. Percentage of total listed for each row. DASS-21 Scale for Anxiety, Depression, and Stress. Moderate and more severe considered “at-risk” for clinical disorder.

### Associations of socio-demographic factors with behavioral health risk factors

According to the results in Table 4, none of the seven socio-demographic factors was meaningfully associated with any of the six behavioral health factors (i.e., all were trivial size statistical effects, see Table 5).

**Table 4:** Tests of associations of student socio-demographic characteristics with behavioral health problems.

**Socio-demographic characteristics**		**Behavioral health problems**			
		**Mental health total**	**Insomnia**	**Drinking**	**Smoking**
**Age**	*r*	0.026	0.011	0.036*	0.03
≤15 years old	*N* (%)	809 (44.6%)	766 (42.3%)	250 (13.8%)	149 (8.2%)
16 years old	*N* (%)	1,697 (44.8%)	1,623 (42.9%)	492 (13.0%)	295 (7.8%)
17 years old	*N* (%)	814 (42.5%)	799 (41.7%)	263 (13.7%)	169 (8.8%)
≥18 years old	*N* (%)	192 (40.5%)	203 (43.0%)	87 (18.4%)	53 (11.2%)
**Sex (0** **=** **male, 1** **=** **female)**	*r*	0.057**	0.093**	0.072**	0.067**
Male	*N* (%)	1,656 (41.1%)	1,523 (37.9%)	647 (16.1%)	410 (10.2%)
Female	*N* (%)	1,856 (46.8%)	1,871 (47.2%)	440 (11.1%)	258 (6.5%)
**Community (0** **=** **rural, 1** **=** **urban)**	*r*	0.019	0.013	0.025*	0.007
Rural	*N* (%)	2,903 (43.5%)	2,811 (42.2%)	886 (13.3%)	546 (8.2%)
Urban	*N* (%)	609 (46.0%)	581 (43.9%)	206 (15.6%)	116 (8.8%)
**Household income per year**	*r*	0.021	0.005	0.066**	0.053**
<1,00,000	*N* (%)	2,880 (44.5%)	2,747 (42.4%)	810 (12.5%)	492 (7.6%)
≥1,00,000	*N* (%)	632 (41.8%)	650 (43.0%)	227 (18.3%)	172 (11.4%)
**Education level—father**	*r*	0.021	0.010	0.040**	0.022
Junior middle school and below	*N* (%)	2,396 (44.7%)	2,279 (42.5%)	729 (13.6%)	456 (8.5%)
High school or technical school	*N* (%)	1,023 (42.5%)	1,012 (42.1%)	315 (13.1%)	185 (7.7%)
College or university and above	*N* (%)	93 (42.1%)	99 (45.2%)	48 (21.7%)	25 (11.3%)
**Education level—mother**	*r*	0.008	0.010	0.039**	0.021
Junior middle school and below	*N* (%)	2,484 (44.2%)	2,392 (42.6%)	741 (13.2%)	461 (8.2%)
High school or technical school	*N* (%)	954 (43.3%)	932 (42.4%)	310 (14.1%)	183 (8.3%)
College or university and above	*N* (%)	74 (43.3%)	66 (39.2%)	38 (22.2%)	21 (12.3%)
**Family type**	*r*	0.021	0.027	0.039*	0.035*
Nuclear family	*N* (%)	1,843 (43.2%)	1,802 (42.3%)	545 (12.8%)	320 (7.5%)
Single parent family	*N* (%)	498 (44.7%)	457 (41.1%)	183 (16.5%)	108 (9.7%)
Reorganized family	*N* (%)	303 (46.0%)	296 (45.1%)	100 (15.2%)	66 (10.0%)
United family	*N* (%)	511 (45.4%)	500 (44.4%)	152 (13.5%)	93 (8.3%)
Other types	*N* (%)	357 (43.0%)	337 (40.6%)	107 (12.9%)	77 (9.3%)

N = 7,984. % is percentage of group at-risk on behavioral health measure. * p < 0.05, ** p < 0.01. All of these correlations were r < 0.10 and thus considered trivial size statistical effects even if significant beyond chance level.

**Table 5:** Summary of statistical effect size results for all correlational analyzes.

	**Behavioral health problems**				
**Socio-demographic characteristics**	**Mental health total**	**Insomnia**	**Drinking**	**Smoking**	
Age	Trivial	Trivial	Trivial	Trivial	
Sex	Trivial	Trivial	Trivial	Trivial	
Community	Trivial	Trivial	Trivial	Trivial	
Household income per year	Trivial	Trivial	Trivial	Trivial	
Education level—father	Trivial	Trivial	Trivial	Trivial	
Education level—mother	Trivial	Trivial	Trivial	Trivial	
Family type	Trivial	Trivial	Trivial	Trivial	
	**Abnormal eating attitudes**				
**Socio-demographic characteristics**	**Dieting**	**Bulimia and food preoccupation**	**Awareness of food contents**	**Compensatory behavior**	**Total scale**
Age	Trivial	Trivial	Trivial	Trivial	Trivial
Sex	**Medium**	**Small**	Trivial	Trivial	**Small**
Community	Trivial	Trivial	Trivial	Trivial	Trivial
Household income per year	Trivial	Trivial	Trivial	Trivial	Trivial
Education level—father	Trivial	Trivial	Trivial	Trivial	Trivial
Education level—mother	Trivial	Trivial	Trivial	Trivial	Trivial
Family type	Trivial	Trivial	Trivial	Trivial	Trivial
**Behavioral health problems**					
Mental health total	**Large**	**Large**	**Large**	**Large**	**Large**
Anxiety	**Large**	**Large**	**Large**	**Large**	**Large**
Depression	**Large**	**Large**	**Large**	**Large**	**Large**
Stress	**Large**	**Large**	**Large**	**Large**	**Large**
Insomnia	**Large**	**Large**	**Medium**	**Medium**	**Large**
Drinking	Trivial	Trivial	Trivial	Trivial	Trivial
Smoking	Trivial	Trivial	Trivial	Trivial	Trivial

Effect sizes: trivial r 0.00–0.09; small r 0.10–0.19; medium r = 0.20–0.29; large r =0.30+. Meaningful effects in bold.

### Scores among CVHHS for abnormal eating attitudes

The average scores of dieting, bulimia and food preoccupation, awareness of food contents, compensatory behavior, and the total scale were presented in Table 2.

### Associations of socio-demographic factors with abnormal eating attitudes

Results determined that very few background factors were associated with the abnormal eating attitude measures (only 3 of 35 tests) (see Table 6). Females had higher levels than males on dieting and bulimia symptoms but not on the other two eating attitude components. All other tests had only trivial size effects (see Table 5).

**Table 6:** Tests of associations of student socio-demographic characteristics with abnormal eating attitudes.

		**Abnormal eating attitudes**				
**Socio-demographic characteristics**		**Dieting**	**Bulimia and food preoccupation**	**Awareness of food contents**	**Compensatory behavior**	**Total scale**
**Age**	*r*	−0.027*	−0.021	0.022*	0.015	−0.019
≤15 years old	*M* (SD)	19.21 (9.03)	8.07 (4.60)	6.49 (3.99)	4.18 (2.74)	37.95 (17.78)
16 years old	*M* (SD)	19.91 (8.86)	8.2 (4.62)	6.76 (4.06)	4.33 (2.77)	38.38 (17.82)
17 years old	*M* (SD)	18.64 (8.77)	7.92 (4.50)	6.67 (3.98)	4.21 (2.67)	37.44 (17.49)
≥18 years old	*M* (SD)	18.62 (9.67)	8 (4.89)	6.97 (4.38)	4.53 (3.15)	38.12 (19.95)
**Sex (0** **=** **male, 1** **=** **female)** ^**a**^	*r*	0.222**	0.138**	0.023*	−0.041**	0.145**
Male	*M* (SD)	17.02 (8.70)	7.46 (4.58)	6.59 (4.24)	4.39 (3.00)	35.47 (18.67)
Female	*M* (SD)	20.98 (8.71)	8.73 (4.55)	6.78 (3.85)	4.16 (2.49)	40.65 (16.63)
**Community (0** **=** **rural, 1** **=** **urban)** ^**a**^	*r*	−0.034**	−0.008	−0.004	0.002	−0.020
Rural	*M* (SD)	18.85 (8.93)	8.07 (4.59)	6.68 (4.06)	4.28 (2.78)	37.89 (17.88)
Urban	*M* (SD)	19.67 (8.92)	8.17 (4.66)	6.72 (4.03)	4.26 (2.69)	38.83 (17.79)
**Household income per year** ^**a**^	*r*	0.002	0.003	0.000	−0.014	0.000
<1,00,000	*M* (SD)	18.98 (8.97)	8.08 (4.61)	6.69 (4.09)	4.3 (2.80)	38.05 (18.03)
≥1,00,000	*M* (SD)	19.02 (8.76)	8.12 (4.59)	6.69 (3.89)	4.2 (2.59)	38.03 (17.17)
**Education level—father**	*r*	0.018	0.008	0.013	−0.007	0.014
Junior middle school and below	*M* (SD)	18.87 (8.85)	8.05 (4.54)	6.66 (4.03)	4.29 (2.75)	37.86 (17.71)
High school or technical school	*M* (SD)	19.14 (9.06)	8.15 (4.71)	6.7 (4.06)	4.25 (2.75)	38.25 (18.05)
College or university and above	*M* (SD)	20.07 (9.40)	8.56 (5.05)	7.1 (4.59)	4.43 (3.15)	40.16 (19.75)
**Education level—mother**	*r*	0.004	−0.008	0.003	−0.005	0.001
Junior middle school and below	*M* (SD)	18.96 (8.89)	8.09 (4.57)	6.69 (4.06)	4.29 (2.77)	38.02 (17.86)
High school or technical school	*M* (SD)	18.99 (8.98)	8.11 (4.67)	6.68 (3.99)	4.26 (2.73)	38.04 (17.81)
College or university and above	*M* (SD)	19.76 (9.29)	7.9 (4.92)	6.84 (4.41)	4.29 (3.03)	38.78 (19.20)
**Family type**		0.015	0.005	0.013	−0.007	0.012
Nuclear family	*M* (SD)	19.07 (8.91)	8.07 (4.59)	6.71 (4.02)	4.28 (2.74)	38.14 (17.74)
Single parent family	*M* (SD)	18.92 (9.09)	8.03 (4.66)	6.72 (4.24)	4.36 (2.89)	38.03 (18.58)
Reorganized family	*M* (SD)	19.07 (8.70)	8.29 (4.77)	6.63 (3.97)	4.22 (2.72)	38.21 (17.56)
United family	*M* (SD)	18.84 (8.77)	8.17 (4.47)	6.47 (3.87)	4.2 (2.66)	37.69 (17.24)
Other types	*M* (SD)	18.74 (9.23)	8 (4.63)	6.85 (4.25)	4.31 (2.89)	37.9 (18.69)

N = 7,984. ^a^point biserial correlation r value. *p < 0.05, **p < 0.01. Correlations r < 0.10 considered trivial size statistical effect even if significant beyond chance level. Effect sizes: small r 0.10–0.19; medium r = 0.20–0.29; large r = 0.30+. Meaningful effects in bold.

### Associations of behavioral health risk factors and abnormal eating attitudes

Results determined that each of the three mental health factors and insomnia were all positively associated with the abnormal eating attitudes total scale and subscales and that all of these associations were large or medium size statistical effects (see Table 7). In contrast, both drinking and smoking status were unrelated to any of the eating measures (all were trivial size effects or not significant, see Table 5).

**Table 7:** Associations of behavioral health problems with abnormal eating attitudes.

	**Abnormal eating attitudes**				
**Behavioral health problems**	**Dieting**	**Bulimia and food preoccupation**	**Awareness of food contents**	**Compensatory behavior**	**Total scale**
Mental health total	0.461**	0.461**	0.345**	0.326**	0.502**
Anxiety	0.452**	0.449**	0.339**	0.321**	0.490**
Depression	0.403**	0.409**	0.307**	0.299**	0.422**
Stress	0.452**	0.449**	0.341**	0.314**	0.490**
Insomnia	0.356**	0.334**	0.235**	0.205**	0.375**
Drinking ^a^	0.028*	0.047*	0.022	0.022*	0.034**
Smoking ^a^	0.003	0.026*	0.003	0.01	0.01

N = 7,984. ^a^point biserial correlation r value. *p < 0.05, **p < 0.01. Correlations r < 0.10 considered trivial size statistical effect even if significant beyond chance level. Statistical effect sizes: small r 0.10–0.19; medium r = 0.20–0.29; large r = 0.30+. Meaningful effects in bold.

**Figure 3:**
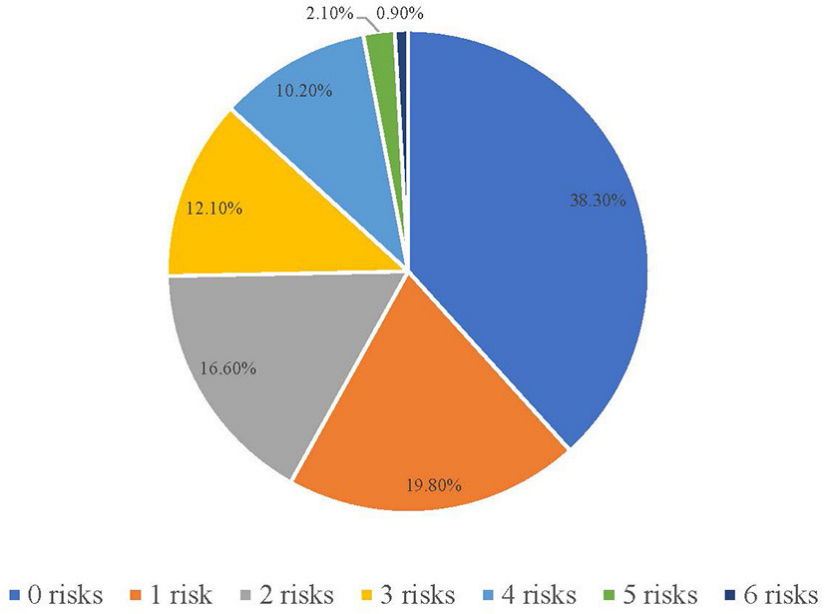
Level of abnormal eating attitudes (EAT total scale score) by number of total behavioral health risks per person among CVHSS.

In addition, the level of abnormal eating attitudes (EAT total scale score) by the number of total behavioral health risks per person is presented in Figure 3. It can be found that the linear trend in the mean scores on the EAT total scale for participants with some of the different levels of the individual level risk summary score. According to the results of the linear trend test, there was a significant association between the number of total behavioral health risks per person and the level of abnormal eating attitudes (*F* = 1,629.46, *p* < 0.001, *r* = 0.412).

## Discussion

This study was designed to investigate the associations between behavioral health risk factors (anxiety, depression, stress, insomnia, drinking, smoking), abnormal eating attitudes and socio-demographic factors among CVHSS in Hunan, China. The findings showed that the prevalence of anxiety, depression, and stress were 41.3, 26.2, and 14.4%, respectively. Of the participants, 43.9% had at least one symptom of depression, anxiety, or stress. A previous study conducted in regular Chinese high schools found that the prevalence of depression and anxiety was 23.8 and 27.2%, respectively, which was lower than what was found in this study (27). It may be attributed to the differences in family relations, academic performance, and social and cultural background between Chinese regular high school students and CVHSS (17). In addition, the prevalence of depression in this study was higher than the prevalence of 7.5% found in Brazil (28), and the prevalence of anxiety was higher than the prevalence of 15.9% found in Italy (29). This suggests that we should pay more attention to the mental health of CVHSS. Previous studies reported that high school students showed a high degree of depression and anxiety in China (71.5 and 54.5%, respectively) (30). In addition, a report released by the Hong Kong Federation of Youth Groups in 2020 showed that more than 52% of students in Hong Kong, China, had a high level of stress (31). The two above-mentioned studies were conducted during the COVID-19 outbreak. Due to fear of infection and lack of contact with friends, students were even more likely to have mental health problems during the pandemic (30).

Compared to Chinese college students, CVHSS scored lower on dieting (18.98 vs. 20.1), bulimia and food preoccupation (8.09 vs. 9.0), awareness of food contents (6.69 vs. 7.2), and the total scale (38.04 vs. 40.1) but higher on compensatory behavior (4.28 vs. 3.8); thus, more attention should be paid to the compensatory behavior of CVHSS. In addition, it can be found that females scored significantly higher on dieting, bulimia and food preoccupation, awareness of food contents, and the total scale than males, consistent with previous findings. Hsu (32) suggested that anorexia nervosa and bulimia nervosa are more common among females than males since females tend to diet to control their weight. Swanson et al. (33) found that females are 2–4 times more likely to report binge eating symptoms than males. Furthermore, in a young Iranian population, females (16.6%) were reported to be more susceptible to extreme dietary restrictions than males (12.0%) (34).

This study found that insomnia was one of the most prevalent risk factors among CVHSS, which may be related to the high academic pressure these students face. Concerns about academic performance keep their minds active at night and make it difficult to fall asleep (35). In addition, some students like to use electronic devices such as computers and smartphones for a long time at night. The light emitted by electronic devices suppresses the melatonin level, which plays a vital role in the effects of sleep in humans (36). Insomnia often leads to listlessness, dizziness, tinnitus, forgetfulness, and neurasthenia during the day, affecting students' academic performance and life (37). To reduce insomnia, school authorities, their families, and other potential sources of support need to create a relaxing environment for students at bedtime while keeping them away from electronic devices and, when necessary, subjecting them to cognitive behavioral therapy (38).

This study demonstrated positive correlations between depression, anxiety, stress, and different types of abnormal eating attitudes. Previous research conducted among various student populations, such as college students (12) and medical undergraduate students (11), also found positive correlations between depression, anxiety, stress, and abnormal eating attitudes. The specific difference between the current study and the above-mentioned research is that, as shown in Table 7, we found that the levels of abnormal eating attitudes were more correlated with anxiety than depression; however, the opposite was found in (12). In addition, our study found that a higher level of insomnia was associated with higher levels of abnormal eating attitudes, consistent with previous findings (39). The potential mechanisms underlying bidirectional associations between them were described in (40). Previous studies also found that other behavioral health problems, such as suicide (41) and non-suicidal self-injury (42) were correlated with abnormal eating attitudes. Future studies should examine the correlations between the above behavioral health problems and abnormal eating attitudes among CVHSS.

Despite the significance of our findings, this study has some limitations. First, all participating CVHSS were from only one province in China (i.e., Hunan) rather than multiple provinces. As economic factors and the admission rate of the National College Entrance Examination may have an impact on high school students' mental health, this could limit the generalizability of our study findings to other provinces. Second, we did not include the reasons for their mental problems in our questionnaire. Finally, participants' mental health symptoms were measured by self-report scales, which may lead to bias compared to diagnostic interviews; future research should consider the use of diagnostic interviews to improve the accuracy of diagnoses.

Our study showed that depression, anxiety, and stress symptoms are common among CVHSS. This suggests that students' mental health requires more attention from school authorities, their families, and other potential sources of support. At present, Chinese vocational high schools have widely launched behavioral health basic education and generally built psychological counseling centers with licensed mental health counselors to provide services to students with behavioral health problems. However, students are often required to seek help proactively; actually, most of them are reluctant. In our study, we found that behavioral health risk factors (anxiety, depression, stress, insomnia, and drinking) were often accompanied by abnormal eating attitude-related symptoms. Therefore, paying attention to CVHSS with abnormal eating attitudes may help detect their mental health problems promptly. The results of this study provide insights for clinical treatment and school-based prevention to improve the mental health of CVHSS. Additionally, with the development of information technology, the use of mobile applications and online tools was considered a promising way to support mental health (43, 44). For example, to increase CVHSS' motivation to seek help, we could sign up for a public account in WeChat (a social software commonly used by the Chinese) to provide mental health knowledge and counseling service.

## Data availability statement

The raw data supporting the conclusions of this article will be made available by the authors, without undue reservation.

## Ethics statement

The study involving human participants was reviewed and approved by the Ethics Committee of Xiangya Second Hospital of Central South University, Hunan, China. Participants over 18 signed a formal informed consent form before participation. In the case of minors, written informed consent was obtained from a parent and/or legal guardian. Written informed consent was obtained from the individual(s), and minor(s)' legal guardian/next of kin, for the publication of any potentially identifiable images or data included in this article.

## Author contributions

JH, RW, ML, and JC conceived the research. JL, ZT, ZC, ZW, TZ, YQ, and HY conducted the research. JL, HT, HX, and HW collected the data. JL and ZT analyzed the results. JL and JH drafted the manuscript. All authors reviewed the manuscript.

## Funding

This study was partially funded by the Key-Area Research and Development Program of Guangdong Province (Grant No. 2018B030334001) and the National Natural Science Foundation of China (Grant Nos. 81971258 and 81901401).

## Conflict of interest

The authors declare that the research was conducted in the absence of any commercial or financial relationships that could be construed as a potential conflict of interest.

## Publisher's note

All claims expressed in this article are solely those of the authors and do not necessarily represent those of their affiliated organizations, or those of the publisher, the editors and the reviewers. Any product that may be evaluated in this article, or claim that may be made by its manufacturer, is not guaranteed or endorsed by the publisher.
