# Supplementary material for: Exploring the associations between behavioral health risk factors, abnormal eating attitudes and socio-demographic factors among Chinese youth: Survey of 7,984 vocational high school students in Hunan in 2020
Source: Front Psychiatry. 2022 Nov 7;13:1000821. doi: 10.3389/fpsyt.2022.1000821 (PMC9676643; doi:10.3389/fpsyt.2022.1000821)
Supplement: Supplementary file 1 [file Data_Sheet_1.PDF]

A1 INFORMED CONSENT

Title of Research: Prevalence, risk factors, and correlations of depression, anxiety, and stress with eating disorders among Chinese vocational high school students

Principal Investigator: Jing Huang, M.D., Ph.D

Purpose of the Research

We are asking you to take part in a research study. The purpose of this research study is to investigate the prevalence, risk factors, and correlations of depression, anxiety, and stress with eating disorders among Chinese vocational high school students. The study principal investigator will discuss with you your responsibilities as a participant. Participants will be recruited at three vocational high schools in Hunan, China. As a participant, you need to answer a self-prepared sociodemographic questionnaire, the 21-item version of the Depression Anxiety Stress Scales (DASS-21), Athens Insomnia Scale (AIS), and Chinese version of the Eating Attitudes Test (EAT-19).

Explanation of Procedures

If you agree to participate, during the course of this study, the following research procedures will occur:

Self-prepared sociodemographic questionnaire

A self-prepared sociodemographic questionnaire including ten dimensions: Need to answer a self-compiled social demographic questionnaire, including age, gender, locality, household income, parents' education levels, whether have colored hair, whether have tattoos, smoking habits, drinking habits, and family type need to be answered.

The 21-item version of the Depression Anxiety Stress Scales (DASS-21)

It includes 21 items consisting of 21 items, 7 items per subscale: depression, anxiety, and stress.

The Athens Insomnia Scale (AIS)

It includes 8 items, of which, the first five items are related to nocturnal sleep and the rest are related to daytime dysfunction.

The Chinese version of the Eating Attitudes Test (EAT-19)

It includes 19 items, and the score range of each item ranges from 1–6 (i.e., “Never” = 1, “Rarely” = 2, “Sometimes” = 3, “Often” = 4, “Very Often” = 5, and “Always” = 6).

All study procedures and testing will be performed under the supervision of qualified personnel.

Questionnaires

There is a small psychosocial risk associated with the completion of questionnaires such as depression or anxiety in responding to some questions.

There also may be risks and discomforts that cannot be foreseen. You will be given more information if other risks are found.

Benefits

You can directly benefit from participating in this research.

The results of this research may help to better understand your situation, but also provide an alternative effective way to understand the mental health problems of Chinese vocational high school students.

Alternatives

The study principal investigator will discuss with you the alternatives to participation and their risks and benefits. The alternative is to not participate in the study.

Confidentiality

Information obtained about you for this study will be kept confidential to the extent allowed by law. However, research information that identifies you may be shared with people or organizations for quality assurance or data analysis, or with those responsible for ensuring compliance with laws and regulations related to research.

Voluntary Participation and Withdrawal

Whether or not you take part in this study is your choice. There will be no penalty if you decide not to be in the study. If you decide not to be in the study, you will not lose any benefits you are otherwise owed.

You are free to withdraw from this research study at any time. Your choice to leave the study will not affect your relationship with this institution. Contact the Principal Investigator if you want to withdraw from the study.

You may be removed from the study without your consent if the sponsor ends the study or if the Principal Investigator decides it is not in the best interest of your health.

Cost of Participation

There will be no cost to you for taking part in this study.

Questions

If you have any questions, concerns, or complaints about the research or a research-related injury including available treatments, please contact the study doctor. You may contact Dr. Jing Huang, by email at jinghuangserena001@csu.edu.cn.

If you have questions about your rights as a research participant, or concerns or complaints about the research, you may contact them at +8615874290980. Regular hours for the OIRB are 8:00 a.m. to 5:30 p.m, Monday through Friday.

Legal Rights

You are not waiving any of your legal rights by signing this informed consent form.

Signatures

Your signature below indicates that you have read (or been read) the information provided above, including your responsibilities as a participant, the alternatives to participation, and the approximate number of subjects involved in the trial. Your signature below further indicates that you agree to participate in this study. You will receive a copy of this signed informed consent form.

Signature of participant	Date
Signature of legal representative	Date
Signature of Person Obtaining Informed Consent	Date

A2 PARENT/GUARDIAN INFORMED CONSENT

Title of Research: Prevalence, risk factors, and correlations of depression, anxiety, and stress with eating disorders among Chinese vocational high school students

Principal Investigator: Jing Huang, M.D., Ph.D

Purpose of the Research

We are asking your child to take part in a research study. The purpose of this research study is to investigate the prevalence, risk factors, and correlations of depression, anxiety, and stress with eating disorders among Chinese vocational high school students. The study principal investigator will discuss with you the responsibilities of a participant. Participants will be recruited at three vocational high schools in Hunan, China. As a participant, your child needs to answer a self-prepared sociodemographic questionnaire, the 21-item version of the Depression Anxiety Stress Scales (DASS-21), Athens Insomnia Scale (AIS), and the Chinese version of the Eating Attitudes Test (EAT-19).

Explanation of Procedures

If you would allow your child to participate, during the course of this study, the following research procedures will occur:

Self-prepared sociodemographic questionnaire

A self-prepared sociodemographic questionnaire including ten dimensions: Need to answer a self-compiled social demographic questionnaire, including age, gender, locality, household income, parents' education levels, whether have colored hair, whether have tattoos, smoking habits, drinking habits, and family type need to be answered.

The 21-item version of the Depression Anxiety Stress Scales (DASS-21)

It includes 21 items consisting of 21 items, 7 items per subscale: depression, anxiety, and stress.

The Athens Insomnia Scale (AIS)

It includes 8 items, of which, the first five items are related to nocturnal sleep and the rest are related to daytime dysfunction.

The Chinese version of the Eating Attitudes Test (EAT-19)

It includes 19 items, and the score range of each item ranges from 1–6 (i.e., "Never" = 1, "Rarely" = 2, "Sometimes" = 3, "Often" = 4, "Very Often" = 5, and "Always" = 6).

All study procedures and testing will be performed under the supervision of qualified personnel.

Questionnaires

There is a small psychosocial risk associated with the completion of questionnaires such as depression or anxiety in responding to some questions.

There also may be risks and discomforts that cannot be foreseen. You will be given more information if other risks are found.

Benefits

Your child can directly benefit from participating in this research.

The results of this research may help to better understand their situation, but also provide an alternative effective way to understand the mental health problems of Chinese vocational high school students.

Alternatives

The study principal investigator will discuss with you the alternatives to participation and their risks and benefits. The alternative is to not participate in the study.

Confidentiality

Information obtained about your child for this study will be kept confidential to the extent allowed by law. However, research information that identifies them may be shared with people or organizations for quality assurance or data analysis, or with those responsible for ensuring compliance with laws and regulations related to research.

Voluntary Participation and Withdrawal

Whether or not you allow your child to participate in this study is your choice. There will be no penalty if you decide not to be in the study. If you decide not to be in the study, you will not lose any benefits you are otherwise owed.

You are free to withdraw from this research study at any time. Your choice to leave the study will not affect your relationship with this institution. Contact the Principal Investigator if you want to withdraw from the study.

Your child may be removed from the study without your consent if the sponsor ends the study or if the Principal Investigator decides it is not in the best interest of their health.

Cost of Participation

There will be no cost to your child for taking part in this study.

Questions

If you have any questions, concerns, or complaints about the research or a research-related injury, including available treatments, please contact the study doctor. You may contact Dr. Jing Huang, by email at jinghuangserena001@csu.edu.cn.

The research team is happy to answer any questions you have about the study. Please contact them at +8615874290980. Regular hours for the OIRB are 8:00 a.m. to 5:30, Monday through Friday.

Legal Rights

You are not waiving any of your legal rights by signing this informed consent form.

Signatures

Your signature below indicates that you have read (or been read) the information provided above, including the responsibilities of participants, the alternatives to participation, and the approximate number of subjects involved in the trial. Your signature below further indicates that you will give permission for my child to participate in this study. You will receive a copy of this signed informed consent form.

Name of participant

Signature of parent and/or legal guardian Date

Signature of legal representative Date

Signature of person obtaining informed consent Date
